# Fostering resilience and post‐traumatic growth in overseas Chinese left‐behind children: The role of autonomy, competence, and relatedness

**DOI:** 10.1002/brb3.70025

**Published:** 2024-09-11

**Authors:** Shengyu Zhao, Ke Zhang, Yingying Lin, Li Han, Chuanjing Liao, Rufang Ye, Meng Na, Syed Shah Alam

**Affiliations:** ^1^ School of Public Basic,Wenzhou Polytechnic Wenzhou Zhejiang China; ^2^ Haicheng Primary School Shenzhen Guangdong China; ^3^ Wenzhou‐Kean University Wenzhou Zhejiang China; ^4^ School of Education Science Mianyang Teacher's College Mianyang Sichuan China; ^5^ School of Education Wenzhou University Wenzhou Zhejiang China; ^6^ School of Fine Arts and Design Wenzhou University Wenzhou Zhejiang China; ^7^ Graduate School of Business Universiti Kebangsaan Malaysia Bangi Selangor Malaysia; ^8^ Department of Marketing, College of Business Administration Prince Sultan University Riyadh Saudi Arabia

**Keywords:** autonomy, competency, left‐behind adolescents, post‐traumatic growth, relational engagement, resilience

## Abstract

**Objective:**

This study examines the impact of parental migration on the psychological well‐being and development of left‐behind children (LBCs) in Zhejiang, China, within the broader context of the country's rural transformations and urban migration. It investigates how intellectual and relational engagement (RE), autonomy (AUT), competence (COM), and relatedness (RES) contribute to resilience (REL) and post‐traumatic growth (PTG) in these children, reflecting on the shift from viewing parental separation merely as a source of trauma to recognizing its potential to foster significant personal growth.

**Methods:**

Utilizing a cross‐sectional design, the research was conducted in April and May 2023 with 1348 LBCs from a total sample of 4049 students inZhejiang. A two‐step random, stratified, cluster‐based sampling strategy was employed, and structural equation modeling was used to examine the hypothesized relationships among the constructs.

**Results:**

The statistical analysis demonstrated significant positive effects of intellectual engagement (IE), AUT, COM, and RE on both REL and PTG (*p* < .05 for all). IE strongly correlated with AUT (*r* = .68, *p* < .001) and COM (*r* = .71, *p* < .001), enhancing REL and facilitating PTG. Additionally, the presence of secure and consistent relationships was identified as crucial for maintaining psychological well‐being, with high correlation coefficients (*r* > .60) underscoring their importance. Notably, REL was found to moderate the relationships among RES, COM, and PTG, highlighting its critical role in the psychological adaptation of left‐behind children.

**Conclusion:**

The study underscores the importance of nurturing intellectual and REs, AUT, and COM to enhance psychological REL and well‐being among LBAs. These elements are crucial for supporting the mental health and developmental needs of children facing the challenges of parental migration. The findings advocate for targeted interventions that can address the unique needs of this vulnerable population, emphasizing the potential for growth and adaptation despite adversities.

## Graphical abstract

This study employs a quantitative research methodology to provide empirical insights into the impacts of parental migration on the psychological well‐being and development of overseas Chinese left‐behind children.

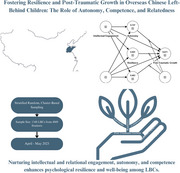

## 1 INTRODUCTION

The evolving landscape of rural China, profoundly altered by economic transformations and rapid urbanization, has given rise to the significant social phenomenon of “left‐behind children” (Du et al., 2020; Liu et al., 2017). These children, estimated at around 9.02 million as of 2016, often find themselves in rural settings, whereas their parents seek employment in urban or overseas locales (Wang, 2022). This demographic shift not only represents a considerable portion of the country's younger population but also brings to the forefront complex challenges concerning their well‐being and development.

Previous research has consistently found that the absence of parental figures in these children's lives contributes to an increased risk of psychological distress, including feelings of abandonment and loneliness (Liu et al., 2020). This heightened vulnerability to stress is a result of their lack of familial support.

Moreover, studies indicate a correlation between parental absence and lower levels of happiness and self‐esteem among these children, potentially impacting their overall life satisfaction (Ai & Hu, 2016). Such emotional and psychological adversities, however, also open avenues for REL and post‐traumatic growth (PTG), underscoring the potential for positive psychological change following hardship (Lan & Wang, 2019).

Although the prevalence of post‐traumatic stress disorder symptoms (PTSSs) in children following traumatic events, including parent–child separation (PCS–PTSS), has been documented (Zhou et al., 2021), there is a growing interest in understanding how these experiences can catalyze PTG. This perspective shifts the focus from the pathology of PTSS to exploring the developmental opportunities that can arise from coping with and adapting to parental absence (Collins, 2022; Kerig, 2017).

In response to the growing concerns regarding the well‐being of left‐behind children in China, significant efforts have been made by the government to address the challenges faced by this demographic (Wang et al., 2020). Central to understanding and supporting these children is the concept of REL, defined as the capacity to improve mental health in the face of traumatic events (Neill & Dias, 2001; Powell et al., 2021). This concept is increasingly being recognized as a pivotal factor in lessening the adverse impacts associated with PTSSs (PCS–PTSS). Research has consistently shown that REL plays a crucial role in reducing the likelihood of developing PTSS or PTSD, and individuals with higher REL are often found to possess more adaptive emotional regulation abilities, which are essential in balancing subjective expectations with actual needs (Anderson & Priebe, 2021; Kim et al., 2017; Zerach & Levi‐Belz, 2019). The emotional flexibility theory further reinforces this notion, suggesting that high REL is linked to the capacity for adaptive emotional response, a vital component in navigating traumatic experiences (Fu et al., 2018). This focus on REL not only sheds light on the psychological dynamics of left‐behind children but also underscores the importance of targeted interventions to bolster their mental health and overall development.

Recent scholarly investigations provide deeper insights into the dynamics shaping the experiences of these left‐behind children. Studies indicate a significant relationship between intellectual engagement (IE) and PTG, where factors, such as social support, emotional intelligence, life events, and parent–child attachment, play mediating roles (Li et al., 2023; Liu et al., 2023; Shek et al., 2023). Furthermore, IE is found to be closely linked with autonomy (AUT), as indicated by research demonstrating its correlation with emotional, cognitive, and behavioral engagement at school and subsequent academic achievement (Wang et al., 2019; Xiani et al., 2019).

Alongside REL, the school environment plays a pivotal role, particularly for left‐behind children in rural areas (Yeung & Li, 2021). According to Bronfenbrenner's ecosystem theory, a child's psychological development is influenced by personal and environmental factors, including the student–teacher relationship, which is a vital component of children's development (Bronfenbrenner, 1999; Eriksson et al., 2018).

In terms of relational dynamics, the significance of IE in fostering relatedness (RES) is highlighted through studies showing its association with self‐esteem, life satisfaction, and school engagement, mediated by parent–child and grand parent–child relationships (Miguel et al., 2021). Additionally, competence (COM) development in left‐behind children, influenced by IE, is underscored by research emphasizing the importance of academic achievement and cognitive skills (Roulleau‐Berger & Liang, 2022; Wang et al., 2023).

To address these gaps, the present study is anchored in a range of psychological theories. Self‐determination theory (Deci & Richard, 2011) is instrumental in understanding how AUT, COM, and RES—fundamental psychological needs—impact the well‐being of these children. Attachment theory (Bowlby & Ainsworth et al., 2013) offers critical insights into the effects of disrupted parent–child relationships, whereas ecological systems theory (Bronfenbrenner, 1999) provides a comprehensive framework for examining the multiple environmental influences on child development. Furthermore, REL theory and the theory of PTG (Aafjes‐van Doorn et al., 2022; Richardson, 2002) focus on the capacity for adaptation and growth following adverse experiences.

Employing a quantitative research methodology, this study is designed to provide empirical insights into the impacts of parental migration on the psychological well‐being and development of left‐behind children. By systematically gathering and analyzing numerical data, the research aims to quantify the extent to which intellectual and relational engagement (RE), AUT, COM, and RES affect the REL and PTG of these children. The use of quantitative methods will enable the identification of patterns, relationships, and potential causal links, thereby offering a robust foundation for the development of targeted policies and interventions. Ultimately, this study endeavors to shed light on the complex psychological landscape of left‐behind children, quantitatively measuring their REL and capacity for growth amidst the challenges posed by parental absence.

The implications of this study extend significantly beyond the academic sphere, offering critical insights for policy‐makers, educators, mental health professionals, and community leaders. By elucidating the complex interplay between intellectual and RE, AUT, COM, and RES, and their collective influence on the REL and PTG of left‐behind children, this research provides a foundation for developing targeted interventions and policies. These findings can inform the creation of supportive educational environments and psychological services tailored to the unique needs of left‐behind children, emphasizing the importance of nurturing AUT, COM, and social connections. Furthermore, the phenomenon of left‐behind children is particularly pronounced in rural China due to its specific socioeconomic conditions, similar situations exist globally, often in regions experiencing rapid urbanization or where economic opportunities necessitate parental migration. For example, in various countries in Africa and Latin America, there has been a documented rise in the number of children left behind due to internal or international migration. By comparing these global instances, we can position our study within a broader context, highlighting the universal relevance of our findings. This comparative approach not only enhances the global appeal of the study but also allows for a more comprehensive understanding of the impacts of parental migration on children across different cultural and socioeconomic backgrounds.

### 1.1 Post‐traumatic growth

The conceptual framework of PTG, first introduced by Tedeschi and Calhoun in the mid‐1990s, evolved significantly by 2018 through their further articulations and those of Kadri et al. (2022). Tedeschi et al. (2018) defined PTG as the positive psychological evolution that occurs through grappling with trauma, suggesting it runs concurrently with, rather than in lieu of, negative psychological impacts. This growth manifests as improvements in self‐perception, relational dynamics, and life philosophy, culminating in increased self‐awareness, confidence, openness, life appreciation, and the exploration of new possibilities (Tedeschi & Calhoun, 1996).

In the broader discourse, life's adversities and fortunes have long been recognized as catalysts for change. Historically, psychiatric research prioritized the pathological impacts of trauma, underscoring its integral role in psychopathological frameworks, focusing primarily on its negative trajectories (Dell'Osso & Carpita, 2023; Dell'Osso et al., 2019). Conversely, the potential positive repercussions of trauma, encapsulated in the emerging interest in “resilience,” have only recently been explored. This shift challenges the earlier notion that negative experiences inevitably result in adverse outcomes, thereby questioning the inevitability of returning to pre‐trauma states (Capaldi et al., 2024; Castiglioni, Caldiroli, Procaccia, et al., 2023).

In this context, recent research sheds light on the specific challenges and potential for REL and PTG among overseas Chinese left‐behind children. Studies have identified critical roles for social support and emotional intelligence in facilitating post‐stress growth (Yuan et al., 2023; Zhang et al., 2023). However, evidence points to a concerning trend where early childhood separation can impede developmental progress, particularly in boys (Zhang et al., 2015). Further research links these early separations to later‐life trauma and mental health issues (Duan et al., 2023). Despite these adversities, certain children exhibit REL, with protective factors such as empathy, initiative, and positive educational expectations emerging as crucial (Castiglioni, Caldiroli, Manzonim, et al., 2023). Nevertheless, pervasive issues, like loneliness, post‐traumatic stress, and depressive symptoms, are common, compounded by the lack of parental supervision and its detrimental effects on educational outcomes (Wang, 2023; Yuan et al., 2023). Additionally, there is an increased risk of severe mental health outcomes, including self‐harm and suicidal ideation among these children (Lin et al., 2024; Wadji et al., 2023). The existing literature, while extensive, indicates a significant research gap: the need for targeted strategies to foster REL and PTG specifically in overseas Chinese left‐behind children.

### 1.2 Integrating psychological theories

This study on the psychological well‐being of overseas Chinese left‐behind children is grounded in a blend of influential psychological theories, each contributing unique insights into the experiences and developmental trajectories of these children. Developed by Deci and Ryan, self‐determination theory (SDT) offers a vital framework for understanding the inherent psychological needs for COM, AUT, and RES, essential for motivation, well‐being, life satisfaction, and vitality. In the context of left‐behind children, whose experiences of parental absence may challenge their AUT, affect their COM, and alter their sense of RES (Lan & Wang, 2022), SDT provides a crucial lens for exploring how these factors interact to impact overall well‐being. Recognized as a metatheory, SDT integrates several mini‐theories (Ryan & Vansteenkiste, 2023), making it a powerful tool for examining the multifaceted experiences of these children and the factors promoting their psychological REL and growth (Ryan & Deci, 2000).

Attachment theory (Bowlby & Ainsworth et al., 2013; Bretherton, 1985) offers critical insights into the psychological effects of disrupted parent–child relationships. This theory is particularly salient for understanding the emotional and behavioral outcomes of children experiencing prolonged separation from their parents (Jones‐Mason et al., 2021). It allows for an in‐depth examination of how early interactions with caregivers shape a child's emotional and social development, providing a basis for understanding the specific challenges faced by left‐behind children (Sun et al., 2015).

Ecological systems theory (Bronfenbrenner, 1979) presents a comprehensive framework for examining the environmental influences on child development. This theory, which categorizes a child's environment into various interrelated systems, helps in understanding how different aspects of their environment, from immediate settings like family and school to broader societal contexts, impact their development (Garbarino, 2017). The theory's emphasis on the interconnectedness of these systems is particularly relevant for exploring the complex realities of left‐behind children (Neal & Neal, 2013).

REL theory (Masten, 2018), focusing on the capacity for adaptation and growth following adverse experiences, is pivotal in understanding how left‐behind children develop coping mechanisms and adaptive skills despite the challenges posed by parental separation (Li et al., 2020). It examines the role of internal strengths and external support systems in fostering REL among these children. Complementing this, the a Growth Perspective on post‐traumatic stress, proposed by Addington et al. (2016), provides a framework for investigating the positive transformations that can occur following traumatic events. This perspective suggests that individuals, including left‐behind children, can experience significant personal growth as a result of their challenging experiences, leading to enhanced personal strength, improved relationships, and a renewed appreciation for life (Tedeschi, 2020).

**FIGURE 1:**
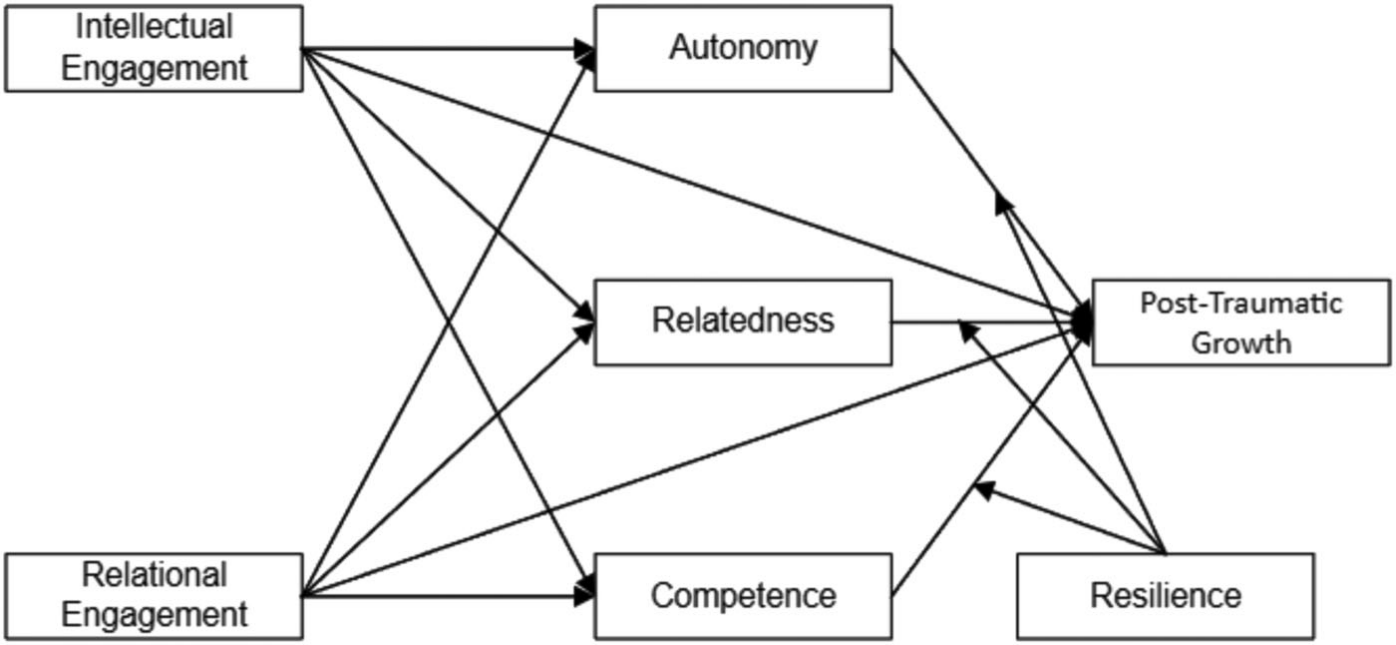
Research framework.

By integrating these theoretical perspectives, our study aims to offer a holistic and nuanced exploration of the experiences of left‐behind children in rural China (Figure 1). It seeks to understand not only their REL in the face of parental absence but also their potential for remarkable psychological growth and adaptation, thus contributing significantly to the academic discourse in this area.

### 1.3 Hypothesis development

#### 1.3.1 Intellectual engagement and its impact on developmental outcomes

Recent empirical studies have begun to unravel the intricate dynamics between IE and PTG among overseas Chinese left‐behind children. Zhang et al. (2022) revealed that factors, such as social support and emotional intelligence, play a mediating role in the relationship between perceived stress and subsequent growth after stress in these children. This finding is consistent with Zhao and Liao (2016), who identified a direct impact of life events on the mental health of left‐behind children, suggesting that external circumstances significantly affect their psychological outcomes. Complementing this perspective, Sun et al. (2015) observed a dichotomy in emotional and educational adjustments of left‐behind children, noting a lower emotional adjustment but an enhanced educational adjustment compared to their non‐left‐behind counterparts. Moreover, Zhang et al. (2022) highlighted the influential role of parent–child attachment and the mediating effects of factors like hometown identity and peer relationships on school adjustment. Together, these studies underscore a complex relationship between IE and PTG, influenced by a constellation of factors, including social support, emotional intelligence, significant life events, and the quality of parent–child attachment.

Building on this understanding, there is substantial empirical evidence linking IE to the AUT of left‐behind children. Xiani et al. (2019) delineated a strong correlation among various forms of school engagement (emotional, cognitive, and behavioral) and academic achievement, a key aspect of AUT. Additionally, Sun et al. (2015) noted heightened school engagement among left‐behind children as compared to their peers, indicating a distinctive trajectory in their academic pursuits. Extending this understanding, Zhang et al. (2022) emphasized how parent–child attachment, mediated by hometown identity, influences school adjustment, a critical factor in developing AUT. Furthermore, Zhou et al. (2009) applied self‐determination theory to demonstrate how autonomous motivation fosters students’ interest, COM, and choice in learning, reinforcing the significant role of IE in nurturing AUT.

The relationship between IE and RES in left‐behind children is also well documented. Xiani et al. (2019) and Song et al. (2018) illustrated the correlation between school engagement and enhanced self‐esteem, life satisfaction, and school engagement, factors pivotal in developing RES. Adding to this narrative, Sun et al. (2015) showed a disparity in emotional adjustment but a superior school engagement among left‐behind children, suggesting a complex relationship between academic involvement and social connectedness. Furthermore, Zhang et al. (2022) elucidated this connection, indicating that parent–child attachment and the influence of social networks, like hometown identity and peer relationships, play a crucial role in fostering RES through school adjustment.

Lastly, a substantial body of research demonstrates a clear relationship between IE and the development of COM in left‐behind children. Studies by Xiani et al. (2019) and Sun et al. (2015) consistently show that emotional, cognitive, and behavioral engagement at school is closely linked with academic success, a marker of COM. Providing further insight, He et al. (2022) noted that cognitive abilities contribute to academic gains, whereas Liu et al. (2018) found that despite lower scores in theory of mind tasks, general reasoning ability helps mitigate the adverse effects of being left behind on cognitive development. These findings collectively underscore:

H1. There is a significant relationship between intellectual engagement and post‐traumatic growth of Overseas Chinese Left‐Behind Children.
H2. There is a significant relationship between intellectual engagement and autonomy of overseas Chinese left‐behind children.
H3. There is a significant relationship between intellectual engagement and relatedness of overseas Chinese left‐behind children.
H4. There is a significant relationship between intellectual engagement and competence of overseas Chinese left‐behind children.

#### 1.3.2 Dynamics among autonomy, relatedness, and competence

Recent literature reveals a profound connection between AUT and PTG in left‐behind children. Zhang et al. (2022) illustrated how social support and emotional intelligence mediate the relationship between perceived stress and subsequent growth, emphasizing the role of AUT in navigating post‐stress adaptation. Complementing this, Zhao and Liao (2016) found a positive correlation between life events and mental health, underscoring the significance of AUT in challenging life circumstances. Further enriching this understanding, Lan et al. (2019) demonstrated that self‐esteem acts as a moderator among stressful life events, depression, and non‐suicidal self‐injury among these children, reinforcing the link between AUT and PTG.

The importance of RES in fostering PTG is also well documented. Zhang et al. (2022) noted that social support can mitigate the negative impacts of perceived stress on growth, highlighting the critical role of RES in the recovery and growth processes. Additionally, Zhao and Liao (2016) showed how life events directly impact mental health, a crucial component of PTG. The influence of parent–child attachment on school adjustment, as described by Zhang et al. (2022), along with observations by Dai and Chu (2018) regarding the negative correlation between being left‐behind and happiness and self‐esteem, collectively emphasize the essential role of RES in fostering PTG among these children.

Furthermore, the literature points to a significant relationship between COM and PTG. Zhang et al. (2022) discovered that perceived stress could negatively impact post‐stress growth, with social support serving as a mediator. Yuan (2022) highlighted that the growth environment significantly influences mental health, encompassing life satisfaction and personal development—key elements of COM. Zhao and Liao (2016) and Zhang et al. (2022) also noted that the role of parent–child attachment in school adjustment supports the notion that COM is a critical factor in facilitating PTG among left‐behind children.

Based on these insights, we propose the following hypotheses:

H5. There is a significant relationship between Autonomy and post‐traumatic growth of overseas Chinese left‐behind children.
H6. There is a significant relationship between Relatedness and post‐traumatic growth of overseas Chinese left‐behind children.
H7. There is a significant relationship between Competence and post‐traumatic growth of overseas Chinese left‐behind children.

#### 1.3.3 The role of relational engagement in promoting growth

Extensive research, including recent findings by Zhang et al. (2022), underscores the pivotal role of RE in facilitating PTG among left‐behind children. This research demonstrates that social support and emotional intelligence significantly mediate the relationship between perceived and growth following stresses. Parent–child attachment, hometown identity, and peer relationships are also influential, impacting school adjustment—an essential aspect of PTG as noted by Zhao and Liao (2016) and Sun et al. (2015).

Further studies highlight the significant link between RE and the AUT of overseas Chinese left‐behind children. Zhang et al. (2022) observed that parent–child attachment significantly influences school adjustment, with hometown identity serving as a mediating factor. Additionally, despite lower emotional adjustment, Sun et al. (2015) found that left‐behind children exhibit greater school engagement compared to their peers. This engagement suggests a robust interface between relational dynamics and AUT. Liu et al. (2020) and Lu (2011) have also commented on the broader effects of RE, noting the impact of remote parental migration on children's psychological REL and the active role of children and their parents in negotiating well‐being.

The importance of relational dynamics is further emphasized by findings that indicate a direct correlation between the quality of these relationships and mental health outcomes. Zhao et al. (2022) reported that left‐behind children with a history of migration experience poorer self‐rated health and a higher risk of depression, highlighting the critical role of interpersonal relationships in mitigating these challenges. Chai et al. (2019) and Lu (2011) identified family functioning and parent–child relationships as key protective factors against loneliness and the broader impacts of parental migration on welfare.

Moreover, academic engagement, which includes emotional, cognitive, and behavioral aspects, has been closely linked to academic success and COM among these children. Xiani et al. (2019) established that these forms of engagement are significantly correlated with academic achievement. This aligns with Zhang et al. (2022)’s observations on the role of parent–child attachment in influencing school adjustment, with hometown identity acting as a mediator. Sun et al. (2015) and Liu et al. (2015) further support this, noting the profound impact of positive teacher–student relationships on emotional and behavioral adjustment, highlighting the protective role of these interactions.

Collectively, these studies reinforce the essential role of RE in enhancing the mental health, AUT, RES, and COM of left‐behind children, leading to the following hypotheses:

H8. There is a significant relationship between relational engagement and post‐traumatic growth of overseas Chinese left‐behind children.
H9. There is a significant relationship between relational engagement and autonomy of overseas Chinese left‐behind children.
H10. There is a significant relationship between relational engagement and relatedness of overseas Chinese left‐behind children.
H11. There is a significant relationship between relational engagement and competence of overseas Chinese left‐behind children.

#### 1.3.4 Resilience as a moderating factor

Research has consistently shown that individuals with left‐behind experiences are more vulnerable to mental health issues, highlighting the critical role of REL in mitigating these effects. Shi et al. (2016) found that promoting REL can help prevent mental health problems in left‐behind children. Further supporting this, Zhao et al. (2020) demonstrated that psychological trait REL fully mediated the relationship between protective factors and self‐esteem/depression in these children. Zhang et al. (2022) added that social support mediated the negative effects of perceived stress on post‐stress growth, with emotional intelligence further moderating this relationship. Similarly, Zhang et al. (2019) identified REL as a protective factor for social adaptation, and Wang et al. (2015) noted that left‐behind children typically have lower self‐concept scores, underscoring potential psychological challenges. Based on these insights, we propose as follows:

H12. Resilience moderates the relationship between autonomy and post‐traumatic growth of overseas Chinese left‐behind children.

Further research by Zhao et al. (2020) reiterates that psychological trait REL plays a central role in alleviating the negative impacts of adverse conditions on left‐behind children's mental health. Zhang et al. (2022) also found that REL could mitigate the adverse effects of perceived stress on post‐stress growth, with social support and emotional intelligence playing intermediary roles. Additionally, Feng et al. (2018) discovered that higher levels of REL were associated with lower anxiety levels among these children. These findings collectively suggest that REL is crucial in overcoming the negative repercussions of being left behind and facilitating PTG. Consequently, we suggest as follows:

H13. Resilience moderates the relationship between relatedness and post‐traumatic growth of overseas Chinese left‐behind children.

Highlighting the broader implications of REL, Shi et al. (2016), Zhao et al. (2020), and Yang et al. (2021) have shown that REL not only mediates the effects of various stressors and teacher–child relationships on psychological outcomes but also significantly moderates how these relationships influence the development of COM and subsequent growth following traumatic experiences. This leads us to propose as follows:

H14. Resilience moderates the relationship between competence and post‐traumatic growth of overseas Chinese left‐behind children.

## 2 RESEARCH METHODOLOGY: CROSS‐SECTIONAL STUDY OF LEFT‐BEHIND CHILDREN IN CHINA

### 2.1 Study design and sampling approach

Our study employed a cross‐sectional design conducted in April‐May 2023, focusing on overseas Chinese left‐behind children (LBCs) in the Zhejiang, known for their significant migrant worker populations, provided a representative sample of the LBC demographic prevalent across China.

We implemented a two‐step random, stratified, cluster‐based sampling strategy. Initially, Zhejiang was randomly selected. Subsequently, ten middle schools and eight high school were chosen randomly. If a selected school had fewer than 200 students, an additional school was selected, resulting in a total of three middle schools and one high school included in our study.

### 2.2 Participant recruitment

All students in the sampled schools were invited to participate in the study. The study materials were distributed to the students in a 30‐min classroom session without the presence of teachers to ensure privacy and minimize potential bias. Of the initial 4574 students approached, 525 either refused participation or returned incomplete questionnaires, resulting in a final sample size of 4049 students (88.52% participation rate).

### 2.3 Identification of Overseas Chinese left‐behind Children

Participants were classified as LBCs or non‐LBCs based on their response to the question, “Did one or both of your parents migrate to another country for work for at least 6 months?”. According to their responses, 1348 students (33.3%) were identified as LBCs (Table 1).

**TABLE 1:** Demographics of the respondents.

Demographic		Count
Age range		10–18 years
Mean age ± SD		15.5 ± 2.2
Gender	Male	644
	Female	704
Grade level	Middle school	897
	High school	451
Separated from	Both parents	964
	Father	367
	Mother	17
Caretaker	One parent	467
	Grandparents	809
	Other	72

The majority (57.27%) were middle school students, whereas 42.73% were high school students, with ages ranging from 10 to 18 years. The gender distribution was nearly equal with 51.05% males and 48.95% females (refer to Table 1). A significant proportion (70.06%) was separated from both parents, whereas 26.00% were separated from their fathers and 3.94% from their mothers. Caregivers for these LBCs included one parent (30.52%), grandparents (62.67%), or other relatives/self‐care (6.81%).

### 2.4 Ethical considerations

The study adhered to the principles of the Declaration of Helsinki and following strict ethical standards. Participation was anonymous, confidential, and voluntary, with informed consent obtained from all participants. There were no biomarkers or tissue samples collected for analysis. Participants had the freedom to withdraw from the study at any point.

### 2.5 Measurement items

For our study on overseas Chinese left‐behind children, we adopted a series of validated measurement items, each tailored to assess specific psychological constructs. For AUT, we utilized items from the AUT subscale of the Psychological Needs Satisfaction in Exercise Scale (PNSES), developed by Wilson et al. (2006). COM was measured using the scale from the same source. IE items were adapted from Salanova et al. (2010) and Tauetsile (2019) Utrecht Work Engagement Scale. The PTG Inventory by Addington (2016) provided items for measuring PTG. RE was assessed using items from the Interpersonal Engagement Subscale of the Student Engagement Instrument developed by Waldrop et al. (2019). REL was measured using items from the Connor–Davidson REL Scale (CD‐RISC) by Bezdjian et al. (2017). Lastly, RES items were based on the RES subscale of the Basic Psychological Needs Scale by Ryan and Deci (2000).

To ensure the cultural and linguistic relevance of these items for the Chinese context, we undertook a rigorous back translation process. Initially, the items were translated into Mandarin by a bilingual expert. Subsequently, another bilingual individual, unaware of the original items, translated the Mandarin version back into English. This process helped in identifying and rectifying any inconsistencies or loss of meaning in translation, ensuring the items’ validity and reliability in the context of Chinese culture and language norms. This back translation method is a widely recognized approach in cross‐cultural research to maintain the integrity of the survey items (Brislin, 1970).

## 3 DATA ANALYSIS

### 3.1 Measurement model evaluation

**FIGURE 2:**
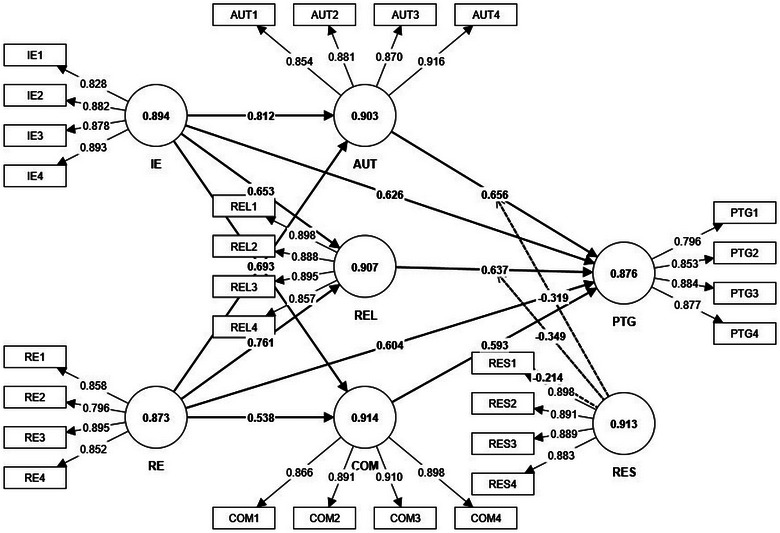
Outer loading, Cronbach's alpha, correlations.

In assessing the measurement model for our study on overseas Chinese left‐behind children, several key statistical tests were conducted, including analyses of Cronbach's alpha, factor loadings, composite reliability (CR), and average variance extracted (AVE) for all constructs (refer to the illustration of Figure 2). We also undertook a detailed analysis of the measurement model to ascertain discriminant validity, a crucial aspect ensuring that our constructs are distinct and not overlapping significantly (Table 2). This analysis involved the Fornell–Larcker criterion, the heterotrait‐monotrait (HTMT) ratio, and a correlation matrix examination, following the methodologies suggested by Hair, Sarstedt et al. (2017).

**TABLE 2:** Result of measurement model and items.

Construct	Code	Items	OL	VIF	CA	CR	AVE
AUT	AUT1	I feel I have the freedom to decide how I live my life	.854	2.243	.903	.903	.776
	AUT2	My choices reflect my true interests and values	.881	2.661			
	AUT3	I feel confident in making my own decisions	.870	2.559			
	AUT4	I am independent in my thoughts and actions	.916	3.527			
COM	COM1	I feel proficient in the tasks I undertake.	.866	2.855	.914	.920	.795
	COM2	I am able to effectively solve problems I face	.891	3.239			
	COM3	I usually succeed in what I set out to do	.910	3.484			
	COM4	I feel competent in handling life's challenges	.898	3.181			
IE	IE1	I am deeply absorbed in my intellectual pursuits	.828	2.056	.894	.895	.758
	IE2	Learning new things is important and exciting to me	.882	2.731			
	IE3	I enjoy discussing and debating ideas	.878	2.599			
	IE4	I engage deeply with tasks that require thinking and analysis	.893	2.832			
PTG	PTG1	I have found personal strengths I didn't know I had due to difficult experiences	.796	1.970	.876	.887	.729
	PTG2	I have changed my priorities about what is important in life after a crisis	.853	2.425			
	PTG3	I appreciate life more after overcoming adversity	.884	2.768			
	PTG4	I have a greater sense of connection to others since experiencing challenges	.877	2.571			
RE	RE1	I actively participate in my relationships	.858	2.316	.873	.879	.724
	RE2	I feel engaged and connected in my social interactions	.796	1.805			
	RE3	I put effort into maintaining my personal relationships	.895	2.783			
	RE4	I feel emotionally invested in my relationships with others	.852	2.063			
REL	REL1	I manage stress effectively	.898	2.944	.907	.908	.783
	REL2	I quickly recover from setbacks	.888	2.759			
	REL3	I am able to adapt to change easily	.895	2.987			
	REL4	I stay determined and focused in face of difficulties	.857	2.277			
RES	RES1	I feel connected and accepted by others	.898	2.878	.913	.914	.792
	RES2	I have close relationships that provide me with support	.891	2.796			
	RES3	I feel a strong sense of belonging in my community	.889	2.846			
	RES4	I feel that my relationships are authentic and meaningful	.883	2.666			

Abbreviations: AUT, autonomy; AVE, average variance extracted; CR, composite reliability; COM, competence; IE, intellectual engagement; PTG, post‐traumatic growth; RE, relational engagement; REL, resilience; RES, relatedness.

The outer loading of the indicators in our study varied between 0.568 and 0.956. Following the guidelines by Hair, Babin et al. (2017), we maintained indicators with outer loadings above 0.50, considering both the statistical significance and content validity of the variables. This decision aligns with the recommendation to keep indicators with a value of more than .40 for further research (refer to Table 2), especially when considering content validity.

We evaluated Cronbach's alpha and CR for all variables. Our findings indicated that both Cronbach's alpha and CR values exceeded .830 and .880, respectively. These results are well above the threshold of .7, as proposed by Hair, Babin et al. (2017), indicating strong internal consistency and reliability of the constructs.

The AVE values for all constructs ranged from .553 to .873, surpassing the minimum requirement of .5. This confirms the convergent validity of our study's constructs, ensuring that they are adequately capturing the variance of the indicators they are intended to measure.

We examined the covariance between the latent exogenous and endogenous constructs. The results demonstrated strong covariance, indicating significant relationships between the constructs. This analysis also revealed the degree of covariance among the exogenous latent variables, providing insights into the interrelationships among the constructs within our study.

The Fornell and Larcker (1981a, 1981b) criterion is a stringent test for discriminant validity, requiring that the square root of the AVE for each construct should be higher than the correlation with any other construct. In our study, this criterion was satisfactorily met. For instance, the construct AUT had an AVE square root of .881, which exceeded its correlations with other constructs like COM at .726 and IE at .812 (refer to Table 3). This pattern was consistent across all constructs, including PTG, RE, REL, and RES, thereby affirming the discriminant validity of our measurement model.

**TABLE 3:** Fornell–Larcker criterion.

	AUT	COM	IE	PTG	RE	REL	RES
AUT	.881						
COM	.726	.892					
IE	.812	.693	.871				
PTG	.656	.593	.626	.854			
RE	.766	.538	.722	.604	.851		
REL	.703	.576	.653	.637	.761	.885	
RES	.640	.748	.619	.700	.497	.460	.890

Abbreviations: AUT, autonomy; COM, competence; IE, intellectual engagement; PTG, post‐traumatic growth; RE, relational engagement; REL, resilience; RES, relatedness.

The HTMT ratio is another approach to assess discriminant validity. Values below the threshold of .85 typically indicate adequate discriminant validity (Henseler et al., 2015). Our results were largely within this acceptable range. For instance, the HTMT ratio between AUT and COM was .794, and between AUT and IE, it was .804, both below the .85 threshold (Table 4). This suggests distinctiveness between these constructs. However, some interaction terms, like RES × REL, showed higher values, necessitating a closer examination to ensure they distinctly capture the intended dimensions of the constructs.

**TABLE 4:** Heterotrait‐monotrait (HTMT) ratio.

	AUT	COM	IE	PTG	RE	REL	RES	RES × REL	RES × COM
AUT									
COM	.794								
IE	.804	.761							
PTG	.727	.647	.696						
RE	.858	.594	.816	.682					
REL	.777	.627	.726	.715	.752				
RES	.704	.817	.684	.771	.553	.505			
RES × REL	.338	.170	.277	.377	.351	.498	.144		
RES × COM	.350	.524	.345	.216	.213	.178	.483	.430	
RES × AUT	.490	.348	.457	.332	.438	.347	.352	.670	.718

Abbreviations: AUT, autonomy; COM, competence; IE, intellectual engagement; PTG, post‐traumatic growth; RE, relational engagement; REL, resilience; RES, relatedness.

The correlation matrix provided further insights into the relationships between constructs. According to Hair, Sarsedt et al. (2017), moderate correlations are expected in behavioral research, but extremely high correlations might indicate redundancy. In our study, the correlations ranged from moderate to high, such as a  .812 correlation between AUT and IE, suggesting significant but distinct relationships (refer to Table 5). These results align well with the theoretical underpinnings of the constructs, confirming that, while related, each construct measures a unique aspect of the psychological experience of left‐behind children.

**TABLE 5:** Correlation matrix.

	AUT	COM	IE	PTG	RE	REL	RES
AUT	1.000						
COM	.726	1.000					
IE	.812	.693	1.000				
PTG	.656	.593	.626	1.000			
RE	.766	.538	.722	.604	1.000		
REL	.703	.576	.653	.637	.761	1.000	
RES	.640	.748	.619	.700	.497	.460	1.000

Abbreviations: AUT, autonomy; COM, competence; IE, intellectual engagement; PTG, post‐traumatic growth; RE, relational engagement; REL, resilience; RES, relatedness.

In summary, the measurement model evaluation for our study indicates robust consistency, reliability, convergent validity, and discriminant validity of the constructs. These results provide a solid foundation for the reliability and validity of the instruments used to measure the psychological constructs of interest in our sample of overseas Chinese left‐behind children.

### 3.2 Hypothesis testing results and discussion

**FIGURE 3:**
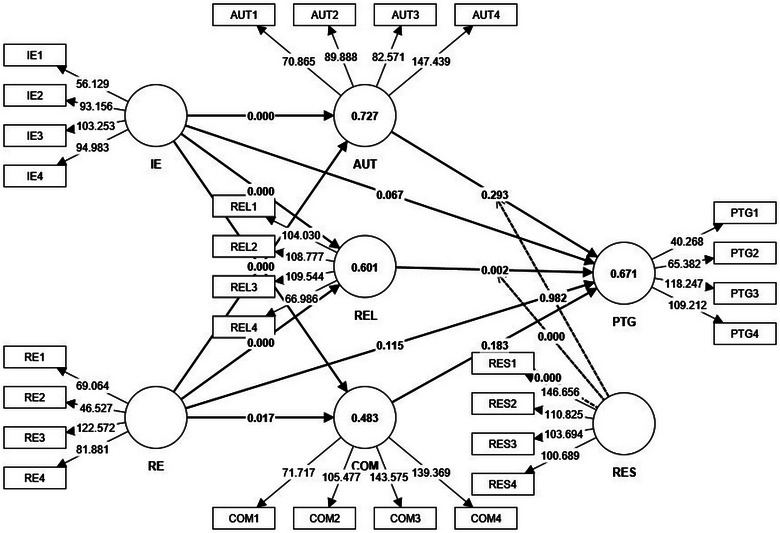
Results of the mediation and moderation model (*R* ^2^, *p*‐value, and *T* statistics).

The study exploring the psychological dynamics of overseas Chinese left‐behind children employed structural equation modeling (illustrated in Figure 3) to test a series of hypotheses linking various constructs such as IE, AUT, COM, PTG, RE, and REL (refer to Table 6). The study's rigorous statistical approach yielded significant and nuanced insights, enhancing our understanding of the intricate psychosocial landscape of these children.

**TABLE 6:** Results of the structural model analysis.

Hypo.	Path	Original sample	Standard deviation	*T* statistics	*p* Values	*f* ^2^	Decision
H1	IE → PTG	0.087	.047	1.829	.067	.006	Not supported
H2	IE → AUT	0.541	.033	16.484	.000	.512	Supported
H3	IE → REL	0.215	.035	6.145	.000	.056	Supported
H4	IE → COM	0.635	.034	18.591	.000	.372	Supported
H5	AUT → PTG	0.048	.046	1.052	.293	.001	Not supported
H6	REL → PTG	0.127	.041	3.139	.002	.013	Supported
H7	COM → PTG	0.053	.040	1.332	.183	.002	Not supported
H8	RE → PTG	0.063	.040	1.577	.115	.003	Not supported
H9	RE → AUT	0.375	.035	10.578	.000	.246	Supported
H10	RE → REL	0.605	.035	17.396	.000	.440	Supported
H11	RE → COM	0.080	.033	2.395	.017	.006	Supported
H12	RES × AUT → PTG	0.001	.031	.022	.982	.000	Not supported
H13	RES x REL → PTG	−0.180	.024	7.355	.000	.077	Supported
H14	RES x COM → PTG	0.189	.026	7.301	.000	.065	Supported

Abbreviations: AUT, autonomy; COM, competence; IE, intellectual engagement; PTG, post‐traumatic growth; RE, relational engagement; REL, resilience; RES, relatedness.

**Hypothesis 1** (IE → PTG) was not supported, with a path coefficient of .087 (*p* = .067). This result aligns with the findings of Zhang et al. (2022) and Zhao and Liao (2016), who noted the significant impact of factors like social support and emotional intelligence on the relationship between stress and PTG. Additionally, Sun et al. (2015) observed a lower emotional but improved educational adjustment in these children. The lack of support for H1 suggests that although IE contributes to certain aspects of growth and adjustment, its direct impact on PTG may not be as pronounced, possibly due to the dominance of factors such as emotional support and life events.

**Hypothesis 2** (IE → AUT) received support with a path coefficient of .541 (*p* < .001). This finding resonates with Xiani et al. (2019), who discovered a strong correlation between school engagement and academic achievement, and Zhou et al. (2009), who emphasized the role of autonomous motivation in learning. These results imply that IE significantly contributes to AUT among left‐behind children, highlighting the crucial role of educational and cognitive experiences in fostering independence and self‐determination.

**Hypothesis 3** (IE → REL) was supported with a path coefficient of .215 (*p* < .001). This positive correlation is corroborated by studies from Xiani et al. (2019) and Song et al. (2018), showing that school engagement is associated with enhanced self‐esteem and life satisfaction. This finding accentuates the importance of IE in improving the quality of social connections and sense of belonging, essential components of RES.

**Hypothesis 4** (IE → COM) was supported with a path coefficient of  .635 (*p* < .001). This support is echoed in the findings from Xiani et al. (2019) and He et al. (2022), who linked IE with academic achievement and cognitive abilities. This suggests that IE is a key factor in developing COM, reinforcing the idea that academic and cognitive efforts play a central role in building self‐efficacy and problem‐solving skills.

**Hypothesis 5** (AUT → PTG) was not supported, indicated by a path coefficient of .048 (*p* = .293). Zhang et al. (2022) and Zhao and Liao (2016) have emphasized the role of AUT in navigating post‐stress adaptation, with mediation from factors like social support and emotional intelligence. Lan et al. (2019) also highlighted the moderating role of self‐esteem in the relationship between stressful life events and mental health outcomes. The lack of support for H5 suggests that although AUT plays a role in challenging life circumstances, its direct influence on PTG may not be as straightforward or significant as other factors, such as social support or emotional REL.

**Hypothesis 6** (REL → PTG) was supported with a path coefficient of; .127 (*p* = .002). Research by Zhang et al. (2022) and Zhao and Liao (2016) indicates that social connections and support are crucial in mitigating the negative impacts of stress and promoting growth. The effect of parent–child attachment on mental health and school adjustment, as explored by Zhang et al. (2022) and Dai and Chu (2018), corroborates our findings, underscoring the significance of RES in the PTG of these children.

**Hypothesis 7** (COM → PTG) was not supported, as shown by a path coefficient of .053 (*p* = .183). Although Zhang et al. (2022) observed that stress affects growth, with COM‐related factors like social support acting as mediators, our study suggests that the direct connection between COM and PTG is less evident. Yuan (2022) and Zhao and Liao (2016) have stressed the importance of the growth environment and mental health; however, these do not seem to directly contribute to a significant impact of COM on PTG in our research.

**Hypothesis 8** (RE → PTG) was not supported, indicated by a path coefficient of .063 (*p* = .115). Although Zhang et al. (2022) and others have highlighted the importance of relational factors like social support and parent–child attachment in post‐stress adaptation and school adjustment, our study suggests that the direct impact of RE on PTG in these children might not be as substantial as previously thought. This could be attributed to the complex interplay of various factors influencing PTG, where RE alone may not be the sole determinant.

**Hypothesis 9** (RE → AUT) received support with a path coefficient of; .375 (*p* < .001). Consistent with the findings of Zhang et al. (2022) and Sun et al. (2015), our study underscores the significant role of RE in shaping the AUT of left‐behind children. Factors, such as parent–child attachment and hometown identity, are crucial in enhancing their school adjustment and AUT, affirming the influential role of RE in fostering independence and self‐determination.

**Hypothesis 10** (RE → REL) was supported, indicated by a path coefficient of .605 (*p* < .001). This finding resonates with the research of Zhang et al. (2022), Zhao et al. (2022), and Chai et al. (2019), emphasizing the importance of RE in RES, particularly concerning mental health and interpersonal relationships. Our study reinforces the notion that RE, including family dynamics and peer relationships, is integral to the psychological and social well‐being of these children.

**Hypothesis 11** (RE → COM) was supported, demonstrated by a path coefficient of .080 (*p* = .017). In‐line with the research of Xiani et al. (2019) and Liu et al. (2015), our findings suggest that various aspects of RE, such as active participation in school and positive teacher–student relationships, are vital for the development of COM. This indicates that RE has a positive influence on the COM of left‐behind children, particularly through mechanisms of educational and social support.

**Hypothesis 12** (RES × AUT → PTG) was not supported, as indicated by a path coefficient of; .001 (*p* = .982). Despite studies by Shi et al. (2016) and Zhao et al. (2020) emphasizing the critical role of REL in addressing mental health challenges, our findings suggest that the moderating effect of REL between AUT and PTG in left‐behind children is not as significant as might be expected. This outcome could be due to the complex interplay of REL with AUT, indicating that other factors might be more influential in driving PTG.

**Hypothesis 13** (RES × REL → PTG) received substantial support, with a path coefficient of −.180 (*p* < .001). This finding corroborates research by Zhang et al. (2022) and Feng et al. (2018), highlighting the pivotal role of REL in amplifying the positive effects of RES on PTG. It suggests that REL significantly strengthens the relationship between RES and the ability of left‐behind children to grow and adapt post‐trauma.

Similarly, **Hypothesis 14** (RES × COM → PTG) was supported, evidenced by a path coefficient of .189 (*p* < .001). This finding aligns with insights from Shi et al. (2016) and Zhao et al. (2020), demonstrating the significant moderating role of REL in the relationship between COM and PTG. It underscores the importance of REL in enabling left‐behind children to develop COM and achieve growth in the aftermath of trauma, highlighting the adaptive power of REL in their developmental journey.

This study offers significant contributions to the field of psychosocial research on left‐behind children. The findings not only enhance our understanding of the psychological impacts of being left behind but also highlight potential areas for targeted interventions and support. The strong influence of IE on key psychological outcomes emphasizes the need for educational and cognitive development initiatives. Simultaneously, the role of REL and RES in fostering PTG opens new avenues for therapeutic and supportive interventions.

In essence, this research sheds light on the multifaceted psychological experiences of overseas Chinese left‐behind children, providing a foundation for future research and practical applications aimed at improving their psychological REL and overall well‐being.

### 3.3 Model fit and predictive relevance

The assessment of our model's fit and predictive relevance has yielded encouraging results, which are instrumental in highlighting its effectiveness in capturing the complexities inherent in the studied constructs (Table 7).

**TABLE 7:** Model fit and internal consistency.

	*R* ^2^	*R* ^2^adjusted	*Q* ^2^predict	RMSE	MAE
AUT	.727	.726	.725	.525	.379
COM	.483	.482	.481	.722	.531
PTG	.671	.669	.576	.653	.458
REL	.601	.601	.598	.636	.434

Abbreviations: AUT, Autonomy; COM, competence; IE, intellectual engagement; PTG, post‐traumatic growth; RE, relational engagement; REL, resilience; RES, relatedness; RMSE, root mean square error.

First, the *R* ^2^ values, indicative of the proportion of variance explained within each construct, were notably high for AUT, PTG, and REL. These values stood at .727, .671, and 0.601, respectively. Such elevated *R* ^2^ values speak to the robustness of our model, demonstrating its capability in explaining a significant portion of the variance within these pivotal constructs (refer to Table 7).

Furthermore, the adjusted *R* ^2^ values exhibited close alignment with the *R* ^2^ values across each construct, further corroborating the reliability of our model. This congruence is vital as it implies that the model's efficacy is retained even when adjustments for the number of predictors are made, thus solidifying the validity of our findings.

Additionally, the model's predictive capabilities were affirmed by positive *Q* ^2^ predict values across all constructs. This aspect is of particular importance as it evidences the model's proficiency in making accurate predictions about the constructs, thereby enhancing its practical applicability in real‐world settings.

Moreover, the accuracy and precision of the model were further validated through the root mean square error (RMSE) and mean absolute error (MAE) values. The RMSE values for AUT, COM, PTG, and REL were recorded at .525,  .722,  .653, and  .636, respectively. Correspondingly, the MAE values stood at  .379,  .531,  .458, and  .434. These metrics are indicative of the model's precision in estimating the constructs, thereby instilling confidence in the reliability of its predictions.

In summary, the analysis has revealed that our model not only demonstrates a good fit but also possesses substantial predictive relevance. These findings are crucial as they underscore the model's efficacy in elucidating the dynamics of AUT, COM, IE, PTG, RE, REL, and RES among overseas Chinese left‐behind children. The model's strengths in both explanatory and predictive dimensions render it a valuable asset in comprehending and addressing the multifaceted needs and challenges encountered by these children.

## 4 IMPLICATIONS OF THIS STUDY

### 4.1 Theoretical implications

The research on the psychological well‐being of overseas Chinese left‐behind children, viewed through the lens of several foundational psychological theories, provides deep theoretical insights. This study has integrated SDT, attachment theory, ecological systems theory, REL theory, and the theory of PTG to understand the complex experiences and developmental trajectories of these children.

SDT, developed by Deci and Ryan, emphasizes the essential psychological needs of COM, AUT, and RES for motivation and well‐being. The study's findings, especially regarding AUT, COM, and IE, align with SDT's principles. The emphasis on IE in fostering AUT and COM resonates with SDT's concept of intrinsic motivation and self‐regulated behavior being crucial for psychological health. This underlines the importance of creating educational and social environments that nurture the AUT and COM of left‐behind children, thus enhancing their intrinsic motivation and REL.

Attachment theory, proposed by Bowlby and Ainsworth (2013), offers crucial insights into the effects of disrupted parent–child relationships. The study's results on RE and its impact on AUT and RES reflect this theory's emphasis on stable relationships for emotional and social development. The findings highlight the need for secure and consistent relationships in developing and maintaining the psychological well‐being of left‐behind children.

Bronfenbrenner's ecological systems theory provides a comprehensive framework for examining the multitude of environmental influences on child development. This theory is exemplified in the study's exploration of the environmental impacts on these children, particularly through the lens of RE's effects on COM and RES. It demonstrates the intricate interplay of various environmental systems in shaping the development of left‐behind children, offering a broad understanding of their complex realities.

Furthermore, the study's findings on REL and its moderating role align with both REL theory and the theory of PTG. The study found that REL significantly moderates the relationship among RES, COM, and PTG. This underscores the capacity of left‐behind children to adapt and grow in adverse conditions, highlighting the role of internal strengths and external support. Additionally, the potential for positive transformation following the traumatic experience of parental separation reflects the essence of the theory of PTG.

In essence, this research enriches our theoretical understanding of the psychological experiences of overseas Chinese left‐behind children. It not only corroborates with established psychological theories but also broadens their scope, providing a detailed understanding of how intrinsic needs, attachment experiences, environmental factors, and REL interact to shape the developmental trajectories of these children. The insights gained are invaluable for guiding future research, interventions, and policies aimed at supporting the well‐being and development of left‐behind children, thus contributing significantly to the academic discourse in this area.

### 4.2 Practical implications

The study on the psychological well‐being of overseas Chinese left‐behind children yields several crucial practical implications, offering guidance for educators, policy‐makers, mental health professionals, and community leaders.

The findings underscore the importance of fostering IE in left‐behind children. Educational programs and curricula need to be tailored to enhance their intrinsic motivation and cognitive development. This can be achieved by integrating more interactive and participatory learning methods, emphasizing critical thinking, and providing opportunities for creative exploration. Additionally, schools should create environments that promote AUT, encouraging students to take initiative and make decisions about their learning process.

The significant role of RE in the development of AUT and COM highlights the need for family support programs. These programs should focus on strengthening parent–child relationships, even in the absence of physical proximity. This could include initiatives like regular communication via digital platforms and providing parents with resources to stay engaged in their children's education and emotional well‐being.

The study's emphasis on the importance of RES and social support points to the need for strong community‐based support systems. Communities can establish mentorship programs where children are paired with responsible adults who can provide guidance, emotional support, and a sense of stability. Additionally, creating community centers where children can engage in group activities, learn new skills, and build social networks could be highly beneficial.

The findings on REL and PTG indicate the necessity for accessible mental health services tailored to the unique needs of left‐behind children. These services should include counseling and therapy sessions focused on building REL, coping strategies for dealing with separation and loss, and programs to enhance self‐esteem and social skills. The study highlights the moderating role of REL in enhancing the effects of RES and COM on PTG, suggesting that interventions aimed at building REL can significantly benefit the psychological well‐being of left‐behind children.

Policy‐makers should consider the study's findings in developing comprehensive child welfare policies. This includes ensuring that educational reforms address the needs of left‐behind children, implementing family reunification programs, and providing financial and social support to families affected by migration. Policies should also focus on creating a supportive ecosystem that encompasses schools, communities, and mental health services, all working collaboratively to support the well‐being and development of these children.

Continuous research and awareness campaigns are essential to keep the issues of left‐behind children in the public discourse. This includes conducting longitudinal studies to track their development over time and understanding the long‐term impacts of being left behind. Awareness campaigns can help in sensitizing the public about the challenges faced by these children, thereby fostering a more supportive and understanding environment for them.

In conclusion, the study's practical implications are multifaceted, addressing educational, familial, community, mental health, and policy aspects. Implementing these recommendations requires a collaborative effort from various stakeholders, ensuring that left‐behind children receive the support and resources they need to thrive both academically and emotionally.

## 5 CONCLUSION AND FUTURE DIRECTIONS

The study on the psychological well‐being of overseas Chinese left‐behind children not only provides valuable insights into their current state but also lays the groundwork for future explorations in this field. The conclusions drawn from this study shed light on the crucial roles of intellectual and RE in shaping the lives of these children. It becomes evident that educational and cognitive experiences significantly influence their sense of AUT, COM, and RES, thus impacting their psychological health. Moreover, the importance of social and familial connections is highlighted, illustrating how RE contributes to their AUT, COM, and RES and, in turn, affects their mental well‐being.

The study also delves into the intricate relationship among REL, RES, COM, and PTG. It uncovers the multifaceted nature of psychological adaptation among left‐behind children, painting a complex picture of their experiences and coping mechanisms. These insights not only enhance our understanding of their current circumstances but also pave the way for future research in this domain.

Looking ahead, there are several promising directions for further research. Longitudinal studies would offer a more in‐depth understanding of the long‐term impacts of being left behind on these children's psychological development. Exploring the experiences of left‐behind children in various cultural contexts or countries could provide a broader perspective and universal insights into this phenomenon. Investigating the effectiveness of different interventions, including educational programs, family support systems, and mental health services, could yield valuable information about the most effective strategies for supporting these children.

Additionally, future research could benefit from examining a wider range of psychological constructs such as emotional intelligence, coping strategies, and social skills. This would provide a more comprehensive view of the needs and challenges faced by left‐behind children. Assessing the impact of specific policies and initiatives aimed at supporting these children could guide policy‐makers in developing more effective strategies. Furthermore, with the advancement of digital technology, exploring the role of technological tools in mitigating the effects of physical separation could offer innovative solutions to some of the challenges faced by these children.

In conclusion, this study marks a significant contribution to our understanding of the lives of overseas Chinese left‐behind children. It highlights critical areas such as IE, relational dynamics, REL, and PTG, providing a foundation for future research and practical interventions. The potential research paths outlined here aim to build on this foundation, advocating for a comprehensive and nuanced approach to this important social issue.

## AUTHOR CONTRIBUTIONS

**Shengyu Zhao**: Writing—original draft. **Ke Zhang**: Formal analysis. **Yingying Lin**: Writing—review and editing. **Li Han**: Conceptualization; funding acquisition. **Chuanjing Liao**: Methodology. **Rufang Ye**: Investigation; validation. **Meng Na**: Writing—review and editing; project administration; data curation. **Syed Shah Alam**: Supervision; software; visualization.

## CONFLICT OF INTEREST STATEMENT

The authors declare no conflicts of interest.

### PEER REVIEW

The peer review history for this article is available at https://publons.com/publon/10.1002/brb3.70025.

## Data Availability

Data will be made available on reasonable request to the corresponding author.
